# Calcium, Phosphorus, and Vitamin D Levels in a Series of Cystic Fibrosis Patients: A Cross-Sectional Study

**DOI:** 10.3390/ijms25031900

**Published:** 2024-02-05

**Authors:** Marlene Fabiola Escobedo-Monge, Marianela Marcos-Temprano, Joaquín Parodi-Román, María Antonieta Escobedo-Monge, Carmen Alonso-Vicente, María Carmen Torres-Hinojal, José Manuel Marugán-Miguelsanz

**Affiliations:** 1 Faculty of Medicine, University of Valladolid, Avenida Ramón y Cajal, 7, 47005 Valladolid, Spain; mctorresh@telefonica.net; 2 Castilla y León Cystic Fibrosis Unit, University Clinical Hospital of Valladolid, Avenida Ramón y Cajal, 3, 47005 Valladolid, Spain; marianela_mt6@hotmail.com; 3 Science Faculty, University of Cadiz, Paseo de Carlos III, 28, 11003 Cádiz, Spain; joaquin_parodi@yahoo.es; 4 Department of Chemistry, Science Faculty, University of Burgos, Plaza Misael Bañuelos s/n, 09001 Burgos, Spain; antoitalia777@gmail.com; 5 Department of Pediatrics of the Faculty of Medicine, University of Valladolid; Section of Gastroenterology and Pediatric Nutrition, University Clinical Hospital of Valladolid, Avenida Ramón y Cajal, 7, 47005 Valladolid, Spain; carmenalonso@gmail.com (C.A.-V.); jmmarugan@telefonica.net (J.M.M.-M.)

**Keywords:** nutritional status, calcium/phosphorus ratio, phosphatase alkaline, physical activity

## Abstract

Cystic fibrosis (CF) is a monogenic disease with different types of mutations that mainly affect the respiratory-digestive system. Calcium (Ca), phosphorus (P), and vitamin D (Vit-D) are essential nutrients for maintaining adequate growth and development, as well as key components in crucial metabolic pathways. Proper diagnosis, treatment, and response are decisive components of precision medicine. Therefore, we conducted a cross-sectional study to evaluate Ca, P, and Vit-D levels along with health and nutritional indicators, regarding their non-skeletal functions, in a series of CF patients. Anthropometric and clinical evaluation, biochemical analysis, dietary survey, and respiratory and pancreatic status were performed. Even though the results showed that all patients had normal dietary and serum Ca levels, 47% of patients had deficient Vit-D intake, 53% of patients had hypovitaminosis D, 35% had insufficient Vit-D levels, 18% had hypophosphatemia, 76% had elevated alkaline phosphate levels, 29% had hypercalciuria, and 65% had hyperphosphaturia. There were no significant differences between homozygous and compound heterozygous patients. Ca, P, and Vit-D levels were associated with body mass index; body composition; physical activity; diet; growth hormones; and the immune, liver, and kidney systems. We suggest a periodically evaluation of Ca and P losses.

## 1. Introduction

Cystic fibrosis (CF) is the most common autosomal recessive multisystem disease worldwide due to the *ΔF508* mutation in gene 7 encoding the CFTR (CF transmembrane conductance regulator) chloride ion channel [1,2,3]. Approximately 2000 mutations in the CFTR gene have been reported [4], affecting 1 in 3300 to 1 in 4800 neonates and 1 in 2500 White individuals [5]. The Cystic Fibrosis Foundation (CFF) Registry data showed a predicted median reported survival of approximately 50 years and revealed that approximately 40% of patients required oral nutrition supplements and 10% received enteral nutrition [6]. Life expectancy has increased the occurrence of other more frequent chronic complications and non-pulmonary comorbidities (non-communicable diseases), along with the increased chance for obesity and overweight, such as CF-related hyperglycemia (diabetes, CFRD), CF-related liver disease (CFLD), CF-related kidney disease (CFKD), and CF-related bone disease (CFBD) [7,8,9].

Cystic fibrosis is a monogenic disease that allows for the evaluation of the phenotype–genotype association (including the response to medications), considering the wide clinical and laboratory spectrum dependent on the interaction between genotype, environment, and lifestyle [1]. Emerging literature suggests that heterozygous CF patients are at increased risk of many of the same conditions as homozygotes, for example. This could cause chronic pancreatitis, infections due to atypical mycobacteria, and bronchiectasis [10]. Whereas personalized medicine is symptom-directed treatment tailored to the patient’s phenotype, precision medicine views the patient as a response to the interrelationship between their environment, lifestyle, and underlying genetics, adjusting conventional drugs according to the individual patient’s dose, the type of the medicine, and the response to the drug [1,11]. Its main objective is to achieve a precise measurement of molecular, environmental, and behavioral factors that contribute to health and disease, in order to obtain an accurate diagnosis, a rational disease prevention strategy, a selection of treatments, and the development of new therapies [11].

Calcium (Ca), inorganic phosphorus o phosphate (P), and vitamin D (Vit-D) are essential components for normal growth and development in human beings [12]. Phosphorus and Ca homeostasis are vital for physiological processes, such as DNA structure, cell signaling, blood coagulation, muscle contraction, bone mineral density, and neuronal excitation [13]. Calcium plays an integral role in neuronal transmission, enzyme activity, myocardial function, coagulation, and other cellular functions [14]. Phosphorus is crucial for the maintenance and repair of all cells and tissues [15]. Vitamin D has anti-inflammatory, insulin-sensitizing effects and regulates Ca and P balance [16]. Hypovitaminosis D is a global health problem leading to many other diseases, most of which are related to a chronic inflammatory state [17]. In CF patients, Vit-D is essential for bone, immune, gastrointestinal (GI) and lung health [18]. At any age, Vit-D deficiency can cause immunodeficiency disorders, with the consequent risk of infectious, as well as the occurrence and progression of autoimmune diseases [19,20].

Not much is known about Ca, P, and Vit-D levels in CF patients concerning their non-skeletal actions. We believe personalized and precision medicine are helpful and indispensable tools to improve the patient’s quality of life with this chronic and debilitating disease. In patients with CF, prophylactic measures, such as altered Ca, P, and Vit-D level detection and monitoring, are necessary to guarantee their adequate nutritional status, growth, and development. In the light of these considerations, we hypothesize that patients with CF may have abnormal levels of Ca, P, and Vit-D associated with other nutrition and health aspects of this chronic disease. Therefore, the main aim of this study was to evaluate Ca, P, and Vit-D levels, regarding their non-skeletal functions, as well as their association with health and nutritional indicators in a series of CF patients. 

## 2. Results

The results of some evaluations carried out on these CF patients have been previously published [2,21,22]. No patients were excluded. The mean age of 17 CF patients (10 females, 59%) was 14.8 ± 8 years (seven children, five adolescents, and five adults). No patients had obesity by body mass index (BMI), but two patients had overweight (8 and 13 years old) and other two had obesity (2 and 25 years old) by waist-for-height index. Although 12% had undernutrition by BMI, no patient had stunted growth. The mean basal energy expenditure (EE) or at rest (REE) was lower than the theoretical one (*p* = 0.001) but was acceptable according to the World Health Organization (WHO)’s recommendation (*p* = 0.074). 

Table 1 summarizes the demographic and clinical characteristics of CF patients and significant differences by mutation types. Homozygous patients had better triceps skinfold; higher intake of polyunsaturated fats, Vit-C, Vit-D and iodine; higher blood urine nitrogen (BUN) levels; and lower levels of transferrin, hemoglobin, leucocytes, platelets, and IgG3 than compound heterozygous ones. The types of *ΔF508* compound heterozygous (65%, 11 cases) are shown in Table 2.

All of 59% of CF patients (10 cases) had respiratory insufficiency (RI; mean score Norman–Crispin 6.3 ± 5.5, forced vital capacity (FVC) 84 ± 38%, and forced expired volume in 1 s (FEV1) 79 ± 26%), and 76% (13 cases) had exocrine pancreatic insufficient (EPI; mean fat-absorption coefficient (FAC) 88 ± 9%). There was no significant difference in weight-for-age (WA), height-for-age (HA), weight-for-height (WH), and BMI Z-score, according to gender and pulmonary function. A lower BMI was observed in those with exocrine pancreatic sufficiency (EPS) compared to the EPI (*p* = 0.020). Lung function was not significantly worse in three patients colonized by *Pseudomonas aeruginosa*, *Candida* spp., and four subjects by *Staphylococcus aureus*, compared to those without such colonization. Although culture-positive patients had normal Vit-D intake, this intake was significantly lower than that of culture-negative patients. Despite their IR and EIP, they carried out daily physical activity (PA). A total of 53% of CF patients (nine cases) played some sport. A total of 59% (10 cases) were between very active/active, three participants did light PA, and four patients were between sedentary/very sedentary.

The nitrogen balance (NB) was positive but lower (mean: 3.7 ± 5.3). The diet was hyperproteic for all CF patients. A total of 47% (8 cases) had a hypercaloric diet; 82% (14 cases) and 59% (10 cases) had a high diet in lipids and cholesterol, respectively; and 35% (6 cases) had a low carbohydrate intake. The diet was deficient in Vit-D (47%, eight cases), Vit-C (41%, seven cases), Vit-A (29%, five cases), zinc (Zn, 23%, four cases), Vit-E (18%, three cases), and magnesium (Mg, two cases). A high consumption of iron (Fe, 88%, 15 cases), Vit-A (53%, 9 cases), Vit-C and E (47%, 8 cases), Ca, Mg, and Vit-D (41%, 7 cases), and Zn (18%, 3 cases) was found. In total, 82% of the subjects (15 cases) had a low Ca/Mg intake ratio. 

Three cases had high C-reactive protein (CRP), and two patients had a high erythrocyte sedimentation rate (ESR). Table 3 displays the age, sex, BMI, dietary Ca and Vit-D intake, the blood and urine tests for Ca, P and Ca/P ratios, serum Vit-D levels and alkaline phosphatase (ALP), and the Ca/Cr (creatinine) ratio, fractional tubular reabsorption of phosphate (TRP) and tubular maximum P reabsorption per glomerular filtration rate (TmP/GFR) of each patient by type of mutation. Four patients had Vit-C deficiency, and five subjects had Fe deficiency. A total of 53% of participants (nine cases) had hypovitaminosis D, and 35% (six cases) had insufficient levels. Only one adult had adequate Vit-D for bone health, and another patient had high levels. There was no association found between Vit-C, Vit-D, Vit-E, and Fe levels and their intake. 

**Figure 1:**
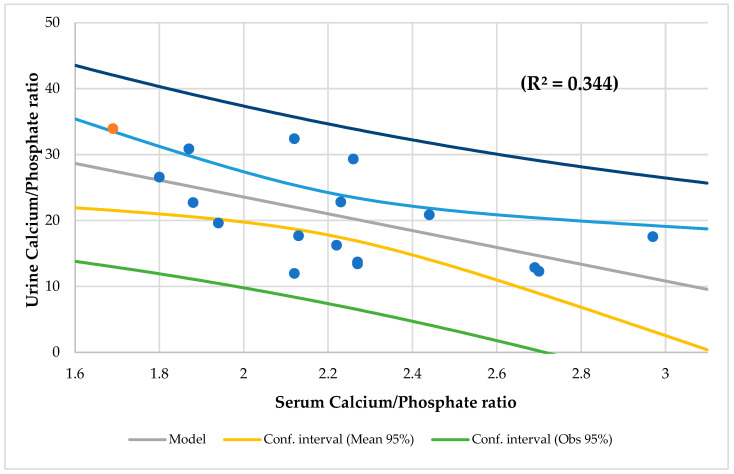
Regression urine calcium/phosphate ratio by serum calcium/phosphate ratio.

**Figure 2:**
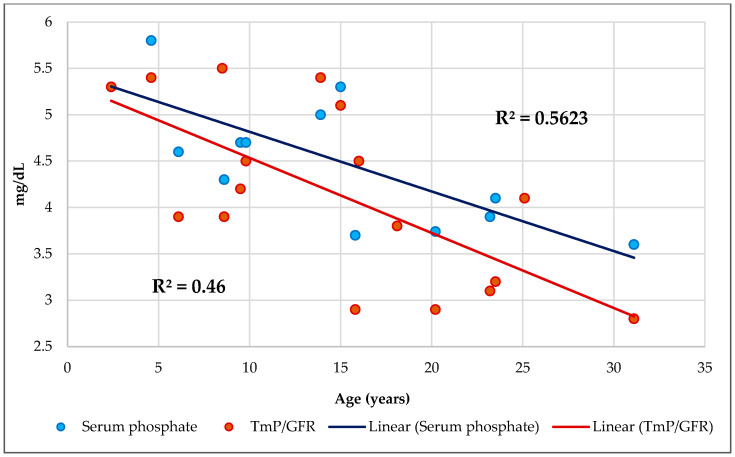
Regression serum phosphate and tubular maximum phosphate reabsorption per glomerular filtration rate (TmP/GFR mg/dL) by age in years.

No association was found between serum Ca and its respective intake. Serum Ca levels were normal. Three patients (18%) had hypophosphatemia, and thirteen cases (76%) had high ALP. The mean serum Ca/P ratio was 2.2. No one children < 18 years of age had an abnormal serum Ca/P ratio, but two subjects ≥ 18 years of age had a lower serum Ca/P ratio. A total of 12% of CF patients had a Ca/Cr ratio > 0.2. Although only four subjects (23%) had a urine Ca loss > 4 mg/kg/day, all patients had a urine Ca loss > 200 mg/L. While 71% of CF patients (12 cases) had a TRP > 86%, 18% (3 cases) had TRP < 85% and only one patient had TmP/GFR < 2.8 mg/dL. A total of 65% of subjects (11 cases) exhibited loss urine P, and 23% of participants (4 cases) had a high TmP/GFR. The mean urine Ca/P ratio was 1.3. There was a negative association between the Ca/P ratios in serum and urine (*R*^2^ = 0.344, *p* = 0.013) (Figure 1). Serum P and TmP/GFR (Figure 2) decrease with age.

Significant correlations between the nutritional parameters studied and the levels of Ca, P, and Vit-D in the diet, blood, and urine are shown in the Supplementary Material (Table S1) (*r* > 0.500 **, *p* < 0.01)]. Table 4 shows the significant associations by simple and multilinear regression analysis (*R^2^* > 0.500 **, *p* < 0.01).

## 3. Discussion

Interestingly, not much is known about Ca, P, and Vit-D levels in CF patients regarding their non-skeletal actions. To the best of our knowledge, this is the first study to explore Ca, P, and Vit-D levels associated with health and nutritional biomarkers in a series of CF patients. The results showed that mean serum Ca level and Ca intake were normal. Even though the diet was hyperproteic, hypercaloric, and rich in lipids and cholesterol, it was low in carbohydrates and deficient in Zn and Mg. The diet was also deficient in Vit-A, D, and E, despite patients receiving pancreatic enzyme replacement therapy (PERT) and fat-soluble vitamin supplements (A, D, E, and K). Nine patients (53%) had Vit-D deficiency, and six cases (35%) had insufficient Vit-D levels. Three participants (18%) had hypophosphatemia, and thirteen patients (76%) had elevated ALP levels. Five patients (29%) had hypercalciuria, and eleven subjects (65%) had hyperphosphaturia. The results show a significant association between Ca, P, and Vit-D with different nutritional and health indicators. 

### 3.1. Clinical Status

In accordance with the present results, 35% of CF patients in our study were homozygous. Previous studies have demonstrated that *ΔF508* is the most common CF-causing mutation, with a prevalence of 30% to 80% depending on the ethnic group [23]. In this study, homozygous patients had better triceps skinfold; higher intake of polyunsaturated fats, Vit-C, Vit-D, and iodine; higher BUN levels; and lower levels of transferrin, hemoglobin, leucocytes, platelets, and IgG3 than compound heterozygous ones. This outcome is contrary to a comparative study between compound heterozygous and homozygous *ΔF508* CFTR pediatric patients. It was observed that the nutritional parameters studied (fat-soluble vitamins, fatty acid profile, weight, height, and BMI) did not differ [24]. Even though genotype did not predict individual nutritional phenotype, based on the molecular phenotypic complexity of CFTR mutants and their susceptibility to pharmacotherapy, mutations may impose combinatorial defects in CFTR channel biology that must be considered [25]. 

Surprisingly, no significant differences were found in WA, HA, WH, and BMI Z-score, according to gender and pulmonary function. A total of 59% of CF patients had RI and 76% had EPI. A lower BMI was observed in those with EPS compared to the EPI (*p* = 0.020). Several investigators have reported that lower fat free mass (FFM) is associated with lower FEV1% predicted and more frequent pulmonary exacerbations in adults with CF [26,27]. In our series, the mean BMI Z-score was normal. Achieving a BMI-for-age above the 50th percentile is a critical goal associated with improved lung function for pediatric CF patients [28]. The results showed that there was a positive association between FFM and fat mass (FM) by anthropometry and bioelectrical impedance analysis (BIA), and both had a positive association with BMI. There were two overweight patients and two obese patients based on waist-to-height ratio. Over-nutrition, in patients with CF, especially those with RI, is a relatively new, emerging phenomenon. Hanna et al. reported that 23% of their patients with CF were overweight or obese [29]. Moreover, in our series, two patients presented undernutrition due to low BMI. Results from a systematic review of 1839 CF patients, including children and adults, found that these patients may be at increased risk of sarcopenia due to their lower FFM, and FM was associated with decreased inspiratory muscle strength [30]. 

Regarding PA, CF patients spent an average of 2.7 h/day in outdoor PA (2 to 5 h/day). Time spent in sports activities had a significant positive correlation with FVC and FEV but not with CAG. FVC and FEV had a strong correlation between each other. Although seven patients in our series (41%) suffered from both RI and EPI, ten of them (59%) were active/very active and played sports. Generally, children with mild CF are more physically active. As in other chronic diseases, inactivity in CF patients may contribute to further reduced exercise tolerance and skeletal muscle dysfunction [31]. Nevertheless, in our series, compound heterozygous patients had more cases with RI and positive cultures, and most of them (71%) were very active/active. In an exercise intervention program conducted on fifty-two CF children, the experimental group had a significant improvement in their exercise capacity, quality of life, and serum Vit-D levels [32]. The PA that our patients developed could have been a key factor in their health status.

### 3.2. Vitamin D

The finding showed that obese and overweight patients (23%) had insufficient levels of Vit-D and that two of them had low dietary Vit-D intake. There is evidence of an inverse relationship between serum Vit-D and FM due to sequestration of Vit-D in fat and volumetric dilution in obese individuals [33,34]. However, in 150 obese children and adolescents, BMI, WH, waist circumference (WC), and FM were significantly inversely correlated with Vit-D levels [35]. In our series, it was the dietary Vit-D intake and urine Ca/P ratio that had a positive and significant correlation with subscapular skinfold thickness. Similarly, in a NHANES 2005–2008 study in 3821 participants (8 to 18 years), subscapular skinfold thickness was the only biomarker of obesity or adiposity that showed significant inverse association with Ca and Vit-D intake. This suggest that this indicator might be useful for cross-sectional and intervention studies [36], as it was in our study. 

This study also found that two patients were malnourished and that despite having a higher dietary intake of vitamin D, they had hypovitaminosis D. CF patients may store less Vit-D due to malabsorption [37]. Decreased body fat, reduced levels of vitamin D-binding protein, and even increased Vit-D catabolism are due in part to CF treatment, e.g., glucocorticoids, antibiotic treatment, among other factors [18]. Compared to 90% of CF individuals with EPI reported in other studies, in our series, thirteen patients (76%) had EPI [37]. Furthermore, in our study, dietary Vit-D intake had a positive correlation with FFM. This result is in line with the current literature, pointing out the possible positive causal effect of serum Vit-D on total appendicular, trunk, and upper body FFM [38]. Moreover, in 116 healthy volunteers (20–74 years), Vit-D had a positive correlation with total lean mass [39]. Studies indicates that Vit-D supplementation increases Ca entry into muscle cells, possibly by regulating the synthesis of cytoskeletal proteins in muscle cells [40].

Even though the diet was rich in lipids (82%), cholesterol (59%), and vitamin D (41%), the diet was deficient in Vit-D in 47% of patients in our series. Curiously, homozygous patients had a higher intake of polyunsaturated fats, as well as Vit-D, than compound heterozygous ones. Vitamin D intake had no significant association with other dietary components or with their serum levels. Data from the 2003–2004 and 2005–2006 National Health and Nutrition Examination Survey (NHANES) on 4404 children aged 2 to ≤19 years showed that serum Vit-D was associated with the Prudent Dietary pattern (all vegetable groups, fruits, other fats, mixed dishes, fish and other shellfish, tomatoes, and meats) but not with the High-Fat–Low-Vegetable Dietary pattern [41]. Nevertheless, in a follow-up study (4 years) developed in 190 Dutch CF children and adolescents, not only was there a significant relationship between total Vit-D intake (dietary and supplementary intake) and serum levels but also between serum Vit-D and pulmonary function. Moreover, while intake remained constant across age/years, serum Vit-D decreased significantly with age [42]. In contrast, in our series, serum Vit-D did not change by age. 

Several reports have shown that it is worrying that in CF patients, Vit-D intake and supplementation do not ensure normal serum levels [43], as seen in our series. In CF patients, Vit-D levels <30 ng/mL were reported between 40% and 90% and <15 ng/mL between 15 and 20% [44]. In our series, median serum Vit-D was in the deficient range, and only one adult had adequate Vit-D levels for bone health. In addition, 35% had insufficient Vit-D levels. Vit-D insufficiency is still a problem in CF patients, even in those receiving supplementations [45]. More than half of our series (53%) had hypovitaminosis D. These results are not surprising because Gupta et al., in 2017, reported that 71% of 52 CF patients (6–18 years) had serum Vit-D levels <15 ng/mL [46]. Timmers et al. noted that 40% of the children had deficient levels and 38% insufficient levels [42]. Furthermore, in our series, two heterozygous adults had severe hypovitaminosis D. Severe deficiency can cause bone malformations in children (rickets) and adults (osteomalacia) [19,47], and it can dramatically increase the risk of mortality, infections, and many other diseases [48]. 

As mentioned in the literature review, the CFF and the ESPEN-ESPGHAN-ECFS guideline has issued instructions for the treatment of Vit-D deficiency, targeting serum levels of at least 30 ng/mL [18,45]. In our series, no patient had serum vitamin levels of 30 ng/mL. Instead of that, four children, two adolescents, and one adult had vitamin D levels between 20.7 and 28 ng/mL. While a concentration of at least 30 ng/mL is adequate for most people to ensure maximum bone health and prevent osteomalacia, a concentration of 40–60 ng/mL is associated with a reduced risk of infectious diseases, cardiovascular disease, neurocognitive dysfunction, and various types of cancer [49,50]. Furthermore, when the Vit-D level is ≥30 ng/mL, the risk of many common cancers is reduced [51]. In our series, an adult had elevated levels of Vit-D (75 ng/mL). Levels >50 ng/mL are too high and can cause health problems [47]. However, Vit-D toxicity requires serum levels ≥150 ng/mL [52]. 

Another significant aspect of Vit-D is a growing body of evidence suggesting that Vit-D plays a role in erythropoiesis and its deficiency may be a factor in the pathogenesis of anemia [53,54]. In our series, homozygous patients had significantly lower hemoglobin, leucocytes, platelets, transferrin, and IgG3 levels than compound heterozygote ones. Serum Vit-D had a positive and significant correlation with mean corpuscular hemoglobin concentration (MCHC), and dietary Vit-D intake had a positive correlation with mean corpuscular hemoglobin (MCH). This result was in line with a NHANES II 2001–2006 study, where in US children and adolescents, hemoglobin levels increased significantly with higher Vit-D quartiles [54]. Similarly, in periodontitis patients, there was a positively correlated between Vit-D and red blood count (RBC) index MCHC [55]. Nevertheless, this outcome is contrary to the KiGGS (Kinder German Health Interview and Examination Survey for Children and Adolescents) study (11 to 17 years), where hemoglobin, MCHC, and RBC were inversely correlated and mean corpuscular volume was positively with serum Vit-D levels [56]. More studies are required to elucidate these facts.

It is key to consider that CF is associated with a hyperinflammatory state [57]. In our series, five patients (29%) had abnormal acute-phase protein activity. Dietary Vit-D intake had a negative correlation with CRP and basophils. Intracellular bacteria are thought to influence cytokine production, and cytokine activation suppresses transcription of the gene 1,25-dihydroxyvitamin-D (1,25(OH) 2D)/Vit-D receptor in monocytes and macrophages [55]. Vitamin D can reduce the proinflammatory cytokine in macrophages and consequently inflammation in the airways, as well as act in the induction of reactive nitrogen and oxygen intermediates or in the induction of autophagy against infections [58]. In addition, CF patients may be at high risk of bacterial infections and worsening of lung function due to Vit-D deficiency because Vit-D can have antimicrobial and anti-inflammatory properties and act as a potent immunomodulator [37]. One patient had a high (copper) Cu/Zn ratio, indicating a possible severe bacterial infection [21,59,60]. Although patients with a positive culture had regular Vit-D intake, this consumption was significantly lower than those with a negative culture. Furthermore, compound heterozygous patients had more cases with positive cultures compared to homozygous ones. Heterozygous carriers of a CFTR variant may be at raised risk of developing bronchiectasis, asthma, allergic bronchopulmonary aspergillosis, and chronic rhinosinusitis [10].

Vitamin D may play a crucial role in preserving lung function in CF patients [18,58,61]. In our series, four patients (23%) with IR had low Vit-D intake. Curiously, although 59% of patients had RI, lung function was not significantly worse in patients colonized compared to those without such colonization. CF patients with Vit-D insufficiency are at risk of pulmonary infection by pathogens (*Pseudomonas aeruginosa*), which can accelerate the decline in lung function. Likewise, in children, hypovitaminosis D is associated with higher rates of pulmonary exacerbation, bacterial colonization, and reduced lung function [18,61,62]. Specifically in CF, locally produced Vit-D can increase LL-37 concentrations to decrease colonization by respiratory tract pathogens [58]. Vit-D produced locally in monocytes or macrophages can act on activated T lymphocytes and activated B lymphocytes, regulating cytokine and Ig synthesis, respectively [51]. What is more, Vit-D deficiency would coincide with a shift from a Th2 to a Th1 immune response [63]. 

The evidence presented in this section suggests that most of our series (88%) presented a high risk of persistent hypovitaminosis D, despite the contribution of PERT and the high intake of Vit-D in some of them, along with their supplementation. This is without forgetting the risk of inflammation and infection secondary to this state of Vit-D deficiency.

### 3.3. Calcium

Prior studies have noted the importance that throughout the world, both developed and less developed countries show insufficient Ca intake [64]. Contrary to expectations, this study did not find any patient with serum Ca deficiency or deficient dietary Ca intake. In contrast, a series of 68 children and adolescents with CF had lower levels of serum Ca [65]. Similarly, in a group of 24 CF adults during a pulmonary exacerbation had a higher prevalence of Ca deficiency [66]. Conversely, in our series, there was no association between serum Ca and its intake. Seven patients (41%) had a high Ca intake, and it was higher in patients with higher Vit-D intake. In contrast, in a study carried on CF patients in Australia, 9.8% of patients did not meet the diet Ca recommendations, and Ca intake was significantly reduced with increasing age groups [67]. This inconsistency may be because Vit-D deficiency would affect the intestinal absorption of Ca [68]. The ESPGHAN recommends a periodic annual assessment of Ca intake, as well as encouraging greater consumption of foods rich in Ca in patients with a suboptimal intake [7,45]. Moreover, the European and French guidelines recommend annual monitoring of calciuria [69]. 

Several reports have shown that a 24 h urine Ca level of 250 mg is an initial threshold for determining hypercalciuria [70]. Even if, in our study, no patients had 24 h urine Ca > 250 mg/L, five cases (29%) had hypercalciuria with regular dietary Ca intake. Two compound heterozygous patients had urine Ca loss > 125 mg/L with high dietary Ca intake The enlarged Ca intake may have been the reason for the increased Ca loss in these two patients. The results showed that in our series, although the mean Ca/Cr ratio was adequate (0.12) [71] and in children and adolescents ranged between 0.02 and 0.10, two homozygous women who had urine Ca loss > 200 mg/L had a high Ca/Cr ratio (0.53 and 0.62) [69], which corroborates hypercalciuria. 

It is essential to keep in mind that malabsorption not sufficiently corrected by PERT, increased intestinal permeability, high-salt diet, or increased endogenous fecal Ca loss and/or high urinary Ca excretion contributes to the negative Ca imbalance [7,69,72]. The findings showed that Ca excretion rate, the most relevant indicator for systematic Ca balance [73], had a positive strong correlation with Ca/Cr ratio, and both decreased with age. In addition, urine Ca had a positive correlation with urine Ca/P ratio and Ca/Cr ratio. Reports demonstrate that due to increased urine Ca loss compared to the general population, CF patients are at higher risk of developing stones at a younger age and require more interventional treatment [74]. Even though in our series patients had hypercalciuria, the normal–high Ca dietary intake may have had a positive contribution to normal serum Ca levels despite the high percentage of patients with EPI and Vit-D deficiency. Although low Ca intake is a risk factor, which our series of patients did not present, Ca may be deficient in people with CF due to Vit-D deficiency [7,69,72], which our series of patients suffer from. 

The results show that NB was positive but minor, even though all patients had a high-protein, high-calorie diet. Negative energy balance resulting from reduced appetite and increased energy requirements contributes to the risk of deteriorating nutritional status in CF patients [30]. Furthermore, the mean basal EE (mean 1077 ± 303 kcal) was lower than theoretical (*p* = 0.001) but was acceptable according to the WHO’s recommendation (*p* = 0.074). The indirect calorimetry (IC) had a positive association with urine P. The mechanisms that explain the increase in REE could be associated with the severity of RI and lung inflammation [30]. A catabolic state induced by low energy intake, insulin deficiency, and chronic inflammation can affect FFM and lead to reduced skeletal muscle mass, inspiratory muscle atrophy, and loss of strength [26]. 

Additionally, in our series, urine nitrogen had an inverse correlation with serum Ca. Higher nitrogen intakes were associated with proportionately higher levels of urinary Ca. Protein intake is a determinant of urinary Ca excretion. Animal protein (which is rich in sulfur-containing amino acids) contributes to an acidic environment, leading to higher excretion of Ca in urine [75]. Furthermore, urine Ca had a positive correlation with Vit-B12 intake, BUN, and gamma-glutamyl transferase (GGT), showing that BUN and GGT together can explain 59% of variability of urine Ca. In menopausal women, 24 h urine Ca levels were correlated with gold standards for assessing Ca absorption, fractional Ca absorption, and net Ca absorption, demonstrating that a urinary Ca level of 24 h < 150 mg/d excluded Ca malabsorption [76].

Another interesting finding in our study was that urine Ca had a negative correlation with basophiles, and that urine Ca/P ratio had a negative correlation with MCHC. RBCs rely on Ca-dependent signaling during precursor cell differentiation. Intracellular Ca levels in circulating RBC participate in the control of biophysical properties (membrane composition, volume, and rheological properties) and physiological parameters (metabolic activity, redox status, and cell clearance) [77]. Basophils are inflammatory cells that originate in the bone marrow from hematopoietic pluripotent stem cells and play an essential role in *Staphylococcus aureus* infection [78]. Furthermore, CF patients exhibit inflammatory overactivation, including increased Ca signaling [79]. Interestingly, CD19 T cells had a negative correlation with urine Ca, and CD3 T lymphocytes had a positive correlation with Ca/Cr ratio and Ca excretion rate. Calcium signaling is crucial for the immune response. In lymphocytes, regulated increases in cytosolic and organellar Ca concentrations control metabolism, proliferation, differentiation, secretion of antibodies and cytokines, and cytotoxicity [80]. In several experimental systems, a hyperinflammatory adaptive immune response dependent on the CFTR genotype of CD3+ CD4+ lymphocytes was demonstrated [81]. 

As far as body composition is concerned, Ca showed an interesting relationship with several assessments. While serum Ca levels had a negative correlation with FM, FFM had a positive correlation with urine Ca and urine Ca/P ratio. Hip perimeter had a positive correlation with urine Ca/P ratio and negative correlation with FFM. Moreover, dietary Ca intake had a positive correlation with suprailiac Z-score. In 355 Spanish college students, dietary Ca intake and muscle strength had an inverse significant association with the FM, suggesting that muscle strength mediates the link between dietary Ca intake and FM percentage [82]. Likewise, Ca may be a key factor for FM regulation, mainly by increasing thermogenesis, reducing lipogenesis, or inhibiting fat absorption [83]. Some studies propose that dietary Ca intake is inverse to adiposity in adults, adolescents, and children [82,84], suggesting that increasing Ca intake could improve body weight/fat loss in children and adolescents, in adults and premenopausal women, or adults over 60 years [85].

Two important themes emerge from the results discussed so far. In our series, all the results analyzed suggest an imbalance in Ca metabolism, due to an increase in urinary and fecal losses despite adequate/high intake and normal serum levels. Furthermore, we should not rule out an imbalance in body composition and the risk of an inadequate immune system response. 

### 3.4. Phosphorus

Phosphorus is one of the most important elements in cellular energy metabolism and acts as a buffer in the blood and urine, contributing to the acid–base balance [86]. Consistent with the literature, this research found that phosphatemia had a negative correlation with age (Figure 2) [87,88,89]. In contrast, serum Ca/P ratio had a positive correlation with age. No CF patient presented hyperphosphatemia, but three patients (18%) had hypophosphatemia. This fact may be due to intestinal malabsorption despite PERT, internal redistribution, and increased urine P losses [68]. Since Vit-D stimulates P absorption by decreasing the parathyroid hormone (PTH) levels and Vit-D deficiency leads to decreased intestinal P absorption, this produces hyperparathyroidism (HPT) and increased PTH-mediated renal P excretion [86]. Chronic diarrhea increases P losses through the intestines and hypovitaminosis D [68]. Furthermore, hypophosphatemia may impair chemotaxis, phagocytosis, and bactericidal activity of macrophages, causing ATP depletion; organ dysfunction; and, especially, muscle weakness. Hypophosphatemic patients with pneumonia had a longer hospital stay with a higher mortality rate [90].

Under physiological conditions, P balance is maintained by fine adjustments of its urinary excretion to equal net GI absorption [91]. The results showed that serum P had a positive correlation with urine P. Eleven participants (65%) had high urinary P losses, indicating risk of hyperphosphaturia [92]. Nevertheless, based on the reference of urine P excretion [93], seven patients in our series (42%) would have hyperphosphaturia. Unexpectedly, the median fractional tubular reabsorption of phosphate (TRP) in our series was normal (78–91%), and no patient had TRP > 95%, which is a marker of P supplement insufficiency [94]. TRP had a negative correlation with IgM. Although twelve subjects (71%) had a TRP > 86% (low P excretion), four patients (23%) had TRP < 85% and had a fractional excretion of P (FEP) > 20%, suggesting kidney damage [95]. There were two homozygous women with high FEP and had low GFR (64 and 61 mL/min/1.73 m^2^). It is important to bear in mind that patients in stages 1–2 of kidney disease, generally asymptomatic, have GFR levels between 60 and <90 mL/min per 1.73 m^2^ [95].

It should be noted that serum Cr may be an unreliable indicator of renal function in CF patients with reduced muscle mass. Kidney damage is often not detected until a significant number of nephrons are affected [96]. An adequate evaluation of GFR can be an important predictive and control factor for kidney damage [97]. In our study, GRF had a positive correlation with serum P, urine P, and TmP/GFR and was negatively correlated with serum Cr and serum Ca/P ratio. Creatinine had a positive correlation with Ca/Cr ratio and Ca excretion rate. When the serum P level increases, the filtered P load increases, and the ability to reabsorb P increases. There is a direct correlation between TmP values and GFR, even when GFR varies over a wide range [98]. Importantly, increased P excretion per Cr clearance may be associated with kidney damage [99].

Additionally, the threshold P concentration in the kidney or TmP/GFR is the main determinant of serum P levels [100]. In addition, Cr had a negative association with TmP/GFR. Phosphorus homeostasis also relies on TRP, ideally assessed by TmP/GFR [101]. In our study, serum P and TmP/GFR had a strong association, and both decrease with age (Figure 2). There was one homozygous 31-year-old woman with a TmP/GFR of 2.8 mg/dL, indicating tubular P leakage. Four patients (23%) had a high TmP/GFR, which indicates renal P wasting. As seen in our series, when TmP/GFR, which also depends on age, decreases in the presence of hypophosphatemia, it indicates renal leakage of P [102]. Chronic hypophosphatemia associated with elevated TmP/GFR can occur in children and is due to impaired intestinal intake or absorption, as in CF [103]. 

Another finding is that, although mean Cr levels were normal, mean BUN levels were high. Homozygous patients had higher BUN levels than heterozygous ones. Two women had high BUN levels, low GFR, and high serum Cr levels with adequate urine Cr. Serum P had a negative correlation with Cr. The Cr-based GFR is a measure of kidney function. The circulating level of Cr is a direct and stable indicator of skeletal muscle mass since its generation is proportional to FFM [104]. In our series, however, the mean BUN/Cr ratio was 27. There were two teenage women and two women with abnormal BUN/Cr ratio (range >60 in children ≤ 10 years and >30 in children > 10 year, adults 10–20) [105]. This ratio may reflect muscle and protein metabolism and the risk of damage to kidney function [106]. 

These results support the notion that this state of hypophosphatemia and hyperphosphaturia may indicate at high risk of P-imbalance metabolism. This likewise highlights the risk of developing kidney damage.

### 3.5. Calcium/Phosphorus Ratios

Calcium and P exert essential roles in many biological processes and may play a positive regulatory role in cell growth and proliferation [107]. In the pediatric stage, they play an indispensable role in their growth and development [12]. Vitamin D regulates Ca and P homeostasis [17], and its deficiency leads to secondary PTH [18,108]. In our series, there were no patients with stunted growth [109], despite 12% having undernutrition based on BMI. While serum P had a negative correlation with HA, serum and urine Ca/P ratio had a positive correlation with HA. It seems possible that these results are due to serum P levels being higher in children than in adults, probably due to the greater requirement to incorporate P into the growing skeleton and soft tissues [107]. We would like to highlight that urine Ca had a low positive association with insulin-like growth factor 1 (IGF-1) and insulin-like growth factor binding protein 3 (IGFBP3), as well as between IGFBP3 with serum Ca and serum P. These findings are consistent with the cross-sectional data from NHANES III, which reported an overall positive correlation between serum Ca and IGF-1 and IGFBP3 levels [110]. 

Concerning Ca and P homeostasis, these nutrients are directly interconnected since serum Ca interacts with serum P by modulating various hormones, so their serum concentration is approximately inversely related [111,112]. In this study, serum and urine Ca/P ratios had a negative correlation between each other (Figure 1). Serum P had an inverse correlation with serum and urine Ca/P ratios. Also, urine P had an inverse correlation with serum and urine Ca/P ratio. Furthermore, serum Ca/P ratio had a negative and significant correlation with TmP/GFR. The mean serum Ca/P ratio was 2.2. Not one child under 18 years of age had an abnormal serum Ca/P ratio, but 40% of subjects ≥ 18 years of age had a lower serum Ca/P ratio, which could be associated with hypoparathyroidism (HypoPT) [113]. In our study, the mean urine Ca/P ratio was 1.3. Very little was found in the literature on the urine Ca/P ratio in human beings. In an animal study (sows), the best cut-off point for urine Ca/P ratio was 1.5 (94% sensitivity and 68% specificity) to identify fed diets deficient in P and 0.5 for diets with an excess of P (sensitivity of 82% and specificity of 82%). If we were based on these values, in our study, five patients (29%) would have diets deficient in P [114]. 

The results of this study show that serum P had a negative correlation with hip circumference, FFM in kg by anthropometry. Similarly, serum Ca/P ratio had a positive correlation WH, hip circumference, FMM in kg by anthropometry, and FM by BIA. Nevertheless, it was the urine Ca/P ratio that had more significant correlations with anthropometric and body composition assessments. It had a positive correlation with WA, wrist, waist and hip circumference, subscapular skinfold, FM and FFM by anthropometry, FFM by BIA, mid upper-arm circumference (MUAC), arm area, arm muscle area, and mid arm muscle circumference (MAMC). These findings are quite surprising given the facts shown by other researchers. In 9202 adults (45 to 100 years), an inverse association was found between serum P and BMI, especially in women, and with the percentage of FM but not FFM, suggesting a causal effect of BMI on serum P [115]. Contrarywise, inverse associations between serum P and BMI, waist-to-hip ratio, WC, and FM were observed in populations with nonmorbid obesity, hypertension, and metabolic syndrome [116,117]. Furthermore, a population-based study included 46,798 South Korean adults without previous comorbidity, finding a negative correlation between serum P with WC and BMI. After adjustment for age, sex, and Ca levels, the association of serum P with WC remained strong, but with BMI it did not remain significant [118].

One unexpected finding was that serum P had a positive and significant correlation with alanine aminotransferase (ALT). Both aspartate amino transferase (AST) and ALT require pyridoxal phosphate as a coenzyme. Two compound heterozygous patients had high levels of AST, ALT, and GGT. AST had a positive and significant correlation with ALT (*r* = 0.835 **, *p* = 0.000). The mean AST/ALT ratio was 1.0 ± 0.2, and eight patients (47%) had an AST/ALT ratio > 1.00. One heterozygous girl (6 years) with normal levels of AST and ALT had an AST/ALT ratio of 1.53, over the upper limit (0.8–1.5) [119]. This may provide some tentative initial evidence that this patient may be at high risk for secondary liver disease. 

Interestingly, serum P had a positive correlation with ALP. ALP had a positive correlation with TmP/GFR and negative correlation with the Ca/Cr ratio, related to losses urinary P and Ca, respectively. The mean ALP was 431 U/L, and thirteen participants (76%) had high ALP levels [120,121]. Two were homozygous women (23 and 25 years) and another was a heterozygous woman (20 years). In general, ALP is 1.5–2.5 times higher in children than in adults [122]. While PA activity in women is highest between 10 and 12 years (ALP of 240 and 400 IU/L), male activity peaks between 13 and 15 years (ALP of 250 and 450 IU/L) [123]. In our series, ALP levels were significantly higher in males (1071 IU/L) than females (555 IU/L, *p* = 0.006). In 341 CF adults, Banjar et al. report that 49% of patients had elevated ALP, 36% had high AST, 56% had elevated ALT, and 23% had high GGT [124]. In 873 cases (1–18 years), the reference range for ALP was 474 to 517 U/L for children 1 to 4 years, 273 to 871 U/L for children 5 to 8 years, 215 to 894 U/L for children 9 to 13 years, and 229 to 739 U/L for teenagers 14 to 18 years [125]. If these cut-off points are considered, patients <18 years old had high ALP and were at risk of developing liver disease. It is important to consider that in CF patients, severe liver disease can appear in mid-childhood (around 10 years old) more so in boys [124]. 

Unexpectedly, in our study, ALP levels had an inverse correlation with age, HA, and kg FMM by anthropometry and by BIA. Bahnemiri et al. found a significant positive correlation between ALP with length (*r* = 0.134), weight (*r* = 0.073), serum P (*r* = 0.122), and ALT *(r* = 0.142). ALP reflects the growth rate in height [125]. Although ALP is elevated due to rickets, it may also be increased in HPT, leukemia, Hodgkin’s lymphoma, congestive heart failure, ulcerative colitis, viral hepatitis, Paget’s disease, fibrous dysplasia, hepatobiliary disease, and during pregnancy [122]. In preterm infants, a serum ALP > 500 IU/L, observed in seven patients (41%) in our series, might indicate osteoporosis (100% sensitivity and 80.77% specificity), being recommended as a reliable biomarker to predict the development of poor bone mineralization [126]. In addition, in our study, Vit-D intake had a negative association with ALP. It is important to consider that the initial stage of osteomalacia related to Vit-D deficiency is characterized by normal serum levels of Ca and P and elevated levels of ALP [127], which occurred in our series.

Another significant aspect of this series was that mean cholesterol intake was slightly elevated, and 59% had high cholesterol intake. Although there was no significant difference in the cholesterol intake by type of mutation, one compound heterozygous female with EPS had cardiovascular risk due to high serum total cholesterol and LDL-cholesterol [128]. Cholesterol intake had a negative correlation with serum P and a positive correlation with serum Ca/P ratio. Although in our series there was no significant difference in total cholesterol, HDL-cholesterol, and triglyceride levels by pancreatic function, EPS patients had significant high LDL levels than PI ones. Cholesterol levels in CF patients are lower than in the general population. Pancreatic endocrine function is what determines cholesterol levels [129]. In a study of 451 CF patients, only 5% had hypercholesterolemia. Patients with PS had between 21 and 36 mg/dL more total cholesterol than PI. This is because patients with PI, even with PERT, have fat malabsorption and chronic inflammation [130]. 

The results reviewed and analyzed here seem to suggest a pertinent role of Ca/P ratios in serum and urine as biomarkers, since both are related to body composition and may suggest an imbalance in Ca and P levels, a state of HypoPT, P-deficient diets, and risk of developing liver disease.

At this point, it is crucial to consider several aspects to highlight. First and foremost, most CF patients (88%) had an increased risk of persistent hypovitaminosis D despite their dietary intake, supplementation, and PERT treatment, showing an alteration in their metabolism. This deficiency state can lead to secondary hyperparathyroidism and changes in the immune system. Secondly, there was an imbalance in the metabolism of Ca and P due to increasing urinary and fecal losses despite their dietary intake. Elevated ALP levels were related to urinary Ca and P losses. We should consider the possibility of an imbalance in body composition and the risk of an inadequate immune system response. Thirdly, the serum Ca/P ratio was associated with the urinary Ca/P ratio, and both may be biomarkers to evaluate Ca and P metabolism. The urinary Ca/P ratio showed associations primarily with anthropometric and body composition assessments. Finally, PA in most of these patients may have contributed to their health and nutritional status. These results should alert us to an increased risk of developing liver and kidney damage, overweight, and obesity, conditions that we can prevent. 

Cystic fibrosis is a chronic disease diagnosed at birth with a longer life expectancy due to improvements in its control and treatment. However, it is a long-term debilitating disease with economy-wide repercussions. We firmly believe that precision medicine and personalized medicine are crucial tools to evaluate all aspects of CF patients’ health status and quality of life. The results respond to the main objective of this study; show their association with health and nutrition biomarkers; and indicate the need to continue studying the relationship between the nutritional status of patients with CF and abnormal levels of Ca, P, and Vit-D, in relation to their non-skeletal functions, to better understand the essential balance between their states. We believe the contribution of our results is valuable, since we show the levels of Ca, P, and Vit-D in each patient and the relationships of these nutrients with other nutritional and health status indicators from a different perspective.

A limitation of this study is the small number of participants and the absence of a healthy control group. Furthermore, an issue we could not address to complete the assessment of these nutrients was the assessment of dietary P intake and Vit-D levels in urine. Nevertheless, notwithstanding the relatively limited sample, this work offers valuable and detailed information on the levels of Ca, P, and Vit-D in this series of CF patients. The strengths of this study lie in the determination of the levels of Ca; P; Vit-D; the Ca/P ratios; and their relationship with anthropometric, biochemical, and dietary indicators, in addition to the evaluation of the health and nutrition status in these patients.

The findings of this study have many practical implications in clinical practice and support the need to evaluate dietary, serum, and urinary Ca, P, and Vit-D levels at least annually. It is important to perform blood and urine studies to confirm that the levels of these nutrients are adequate to make the adequate changes in their diet or to add supplements. Further studies, which take these variables into account, will need to be undertaken. A natural progression of this work is to implement multicenter trials to improve knowledge of Ca, P, and Vi-D to determine the necessary and appropriate amount of supplementation for effective prevention with personalized nutritional recommendations.

## 4. Materials and Methods

### 4.1. Study Site, Design, and Participants

**Figure 3:**
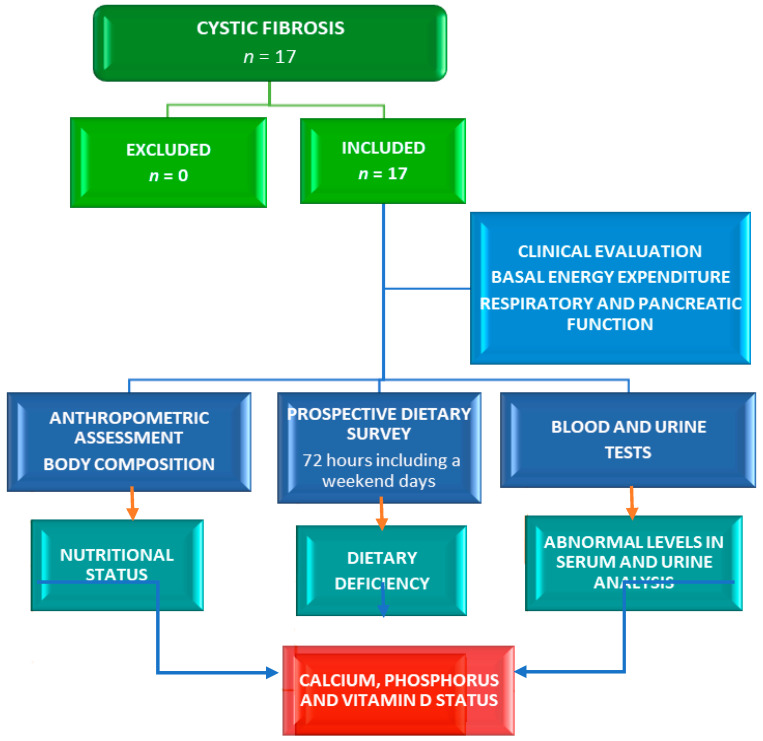
Flowchart of a cross-sectional study design to evaluate calcium, phosphorus, and vitamin D status in a series of cystic fibrosis patients (*n* = 17).

Calcium, P, and Vit-D levels were studied in a series of child, adolescent, and adult CF patients. The design of this cross-sectional and observational study has been previously described (Figure 3) [2,21,22]. This study was carried out for 18 months in the entire population diagnosed with CF that was treated in the Nutrition Unit of the Pediatric Service of the University Clinical Hospital of Valladolid, a reference center in the Community of Castilla y León, Spain. In patients over 15 years of age (adults) who were recruited, the Nutrition Unit continued to monitor the nutritional status and control the treatment in these patients. Patients with a proven diagnosis of CF were included. Patients with acute infection, hospitalization, and refusal to participate were excluded. 

After consulting the project with the clinical research ethics committee of the center, researchers considered that it was not necessary to have a control group and perform unnecessary blood extractions in healthy children. All the biochemical determinations performed are routine studies in clinical practice with previously validated reference values for this population in the clinical analysis laboratory of the hospital, but for the previously published determinations of serum Zn [21] and Cu [22] carried out in the Department of Chemistry of the University of Valladolid. 

### 4.2. Ethical Consideration

The ethics committee of the University Clinical Hospital in Valladolid approved the study protocol (INSALUD-Valladolid, 14 February 2002). It was carried out in accordance with the recommendations of the Declaration of Helsinki. Written informed consent was obtained from all patients prior to their participation in the study. 

### 4.3. Clinical Evaluation

Demographic data were collected from a questionnaire that included the type of mutations *ΔF508*. We divided the series into two groups according to genotype: the homozygous genotype for two identical CFTR pathogenic variants and the compound heterozygous genotype for two different CFTR pathogenic variants. The Norman–Crispin score (>5), FVC% (<80%), and FEV1 (<80%) predicted value by spirometry to estimate RI or RS and the FAC (>93%) [131] by 72 h quantitative fecal fat collection to value EPI or EPS were realized. Daily PA was assessed using a questionnaire adapted to the age groups (children, adolescents, adults) studied, based on the Global Physical Activity Questionnaire (GPAQ) [132]. We gathered data on PA during the week prior to blood and urine sampling, covering type and time spent in occupational PA (school/work), PA at home, and recreational PA (sports/other extracurricular activities). PA was divided into three categories: very active/active, light, and sedentary/very sedentary. 

### 4.4. Assessment of Phenotypical Characteristics

Anthropometrics were assessed using standard weight, height, wrist, hip, waist, and mid-arm circumference techniques. Using Frisancho [133] and Orbegozo tables [134], the Z-score of WA, HA, age-for-50°height, WH, BMI-for age, BMI-height-age, the MUAC and MAMC, FFM, and FM were calculated. Using a Holtain Skinfold Caliper, triceps, biceps, subscapular, and suprailiac skinfolds thickness were measured. The BMI-for-age Z-score was used to categorize patients as underweight (<−2 standard deviations (SD)), normal BMI (−2 to +2 SD), or obese (>+2 SD). Body composition assessed three compartments, namely, FM, bone mineral content, and FFM, using anthropometric measurements and bioelectrical impedance analysis (BIA) (RJL BIA-101 (RJL System, Detroit, MI, USA)). Basal EE or at REE was measured with IC in fasting, using a canopy system under standardized conditions (Deltarac II (Datex-Ohmeda. Helsinki, Finland)). 

### 4.5. Dietary Assessment

Patients/parents/guardians recorded food consumption (all foods consumed) and their quantities as measured by household in a 72 h prospective dietary survey (including one of the weekend days) on the week prior to the blood test. Daily energy intake; fiber; carbohydrates; protein; lipids; monounsaturated, polyunsaturated, and saturated fats; Vit-A, Vit-B1, Vit-B2, Vit-B6, Vit-B12, Vit-C, Vit-D, Vit-E, niacin, and folic acid; and Ca, Mg, Fe, Zn, and iodine were calculated as the percentage of %DRI or adequate intake using the Mataix Food and Health software, which provided the percentage of actual nutrient intake relative to the Spanish recommendations [135,136]. The normal range of dietary intake was 80% to 120% DRI. For their underlying disease, patients received PERT and fat-soluble vitamins (A, D, E, and K) supplements. NB was evaluated as follows: NB (g/24 h) = N supplied (diet) − (total urinary N in 24 h + extrarenal N losses).

### 4.6. Laboratory Exploration

Fasting venous blood samples were collected. Complete blood count, biochemical profile, and acute-phase protein activity, including CRP > 4 U/L and ESR in women >20 mm/h and men >15 mm/h, were measured using standardized methods. Serum prealbumin ≤18 mg/dL, albumin ≤3.5 g/dL as visceral protein reserve, transferrin ≤200 mg/dL, lymphocytes <2000 cell/mm^3^, total cholesterol >200 (mild-moderate risk) and >225 mg/dL (high risk), and low-density-lipoprotein cholesterol >115 (mild-moderate risk) and >135 mg/dL (high risk) were used as cutoffs to evaluate abnormal values. Total immunoglobulin (Ig) G levels of IgG1-4, IgA, IgM, and IgE; complement C3 and C4, CD3, CD4, CD8, CD16+56, CD19 T lymphocytes, and CD4/CD8 ratio; IGF-1 and IGFBP3; folic acid; beta-carotene; vitamins B12, C, D, and E; and Ca, P, Fe, and Mg [2] were measured using standardized methods. Serum Zn [21] and Cu [22] levels were measured by atomic absorption spectrophotometry. 

For serum evaluations, the following cut-off points were used: Vit-D in children: severe deficiency <5 ng/mL, deficiency < 20 ng/mL, insufficiency 20–30 mg/mL, sufficiency >30 ng/mL [137,138]; in adults: adequate for bone health >20 ng/mL, inadequate <20 ng/mL, <12 ng/mL insufficient, >50 ng/mL high levels [47]. Serum Ca in children: 8.8–10.8 mg/dL; in adults: 8.4–10.6 mg/dL [139]; and hypercalcemia >11 mg/dL [140]. Serum Ca was corrected when the serum albumin level was <4.0 g/dL, using the formula: Corrected Ca (mg/dL) = Measured Ca (mg/dL) + [4 − albumin (g/dL) [141]. Serum P in children was 4.5–6.5 mg/dL, in adults was 3.0–4.5 mg/dL [139]. The serum Ca/P ratio < 18 y 2.2 ± 0.5 and >18–34 y 2.7 ± 0.5. Male 1.6–4.4 and female 1.8–3.9 [142]; >3.5 for primary hyperparathyroidism (PHPT) [112], and <2.3 (normal range from 2.3 to 3.3 for HypoPT) [113]. ALP in children from 1 to <10 y 156–369 U/L, from 10 to <13 y 141–160 U/L, from 13 to <15 y, female: 62–280 U/L, male: 127–57 U/L; from 15 to <17 y, female: 54–128 U/L, male: 89–365 U/L; from 17 to <19 y, female: 48–95 U/L, male: 59–164 [120]; and in adults: 30–120 U/L [121]. 

In 24 h urine, urine Ca, P, and Cr were evaluated by standardized methods, using the following cut-off points: hypercalciuria in children > 4 mg/kg/d [143] and >200 mg/L (20 mg/dL), in adults >350 mg/24 h [71]. Hypercalciuria by Ca/Cr ratio from 2 to 4 y: >0.28, children ≥ 4 y > 0.20 (<0.20 is normal while less than 0.18 mg Ca/Cr is optimal) [70]. Urine P: 12.4 ± 4.6 mg/kg/d [92]. The TRP {1 − [(urinary P × serum Cr)/(urinary Cr × serum P)]} × 100 [144]: 78–91% [92]. TmP/GFR: If TRP ≤0.86 then TmP/GFR = TRP × serum P. If TRP >0.86, then TmP/GFR = α × serum P, where α = 0.3 × TRP/{1 − (0.8 × TRP)} [88]. TmP/GFR: from 1 to 5 y 3.25–5.51 mg/dL, from 6 to 12 y 3.00–5.08 mg/dL, from 13 to 15 y 2.82–5.20 mg/dL, and ≥16 y and adults 2.60–3.80 mg/dL [145]. TmP/GFR <2.8 mg/dL indicated the renal tubular phosphate loss [88]. 

### 4.7. Statistical Analysis

All data were recorded in a database. The main variables studied were serum levels of Ca, P, and Vit-D; urinary levels of Ca and P; and dietary intake of Ca and Vit-D. Secondary variables were clinical and anthropometric evaluations, complete blood count, blood and urinary biochemistry, diet, body composition, and baseline EE. The distribution of anthropometric results (quantitative and Z-scores) and biochemical data were described as mean, median, quartiles, standard deviation, and ranges. Disease duration was shown in months. The deficient state of the biomarkers was studied, and the comorbidities were expressed as percentages. Patients were categorized into *ΔF508* homozygous and compound heterozygous. A comparison between groups for continuous and categorical variables was performed using the Mann–Whitney U and McNemar’s tests, respectively. Spearman’s correlation was performed to test the associations. The analysis of variance (Kruskal–Wallis test) was used to search for interactions. To determine if two qualitative variables were independent, we used Fisher’s exact test (FET). To estimate the magnitude of the association between exposure and disease, we calculated odds ratios (ORs). Simple and multiple linear regression analyses was calculated to study the relationships between two or more correlations. The effects were analyzed using the forward method, including only variables with a p-value <0.05. Only correlations/associations with a significant association >0.500, *p* < 0.01 ** are be shown. Analyses were carried out using IBM SPSS version 26.0 (IBM Corp., Armonk, NY, USA). *p*-values < 0.05 * and < 0.01 ** were considered statistically significant. 

## 5. Conclusions

In this series of CF patients, although dietary intake and serum calcium were normal and no patient had hypo- or hypercalcemia, there were patients with hypercalciuria (29%). Even though some patients had hypophosphatemia (18%), most participants had elevated alkaline phosphatase levels (76%) and hyperphosphaturia (65%). Our patients were at increased risk of vitamin D deficiency (88%) despite high dietary vitamin D intake, supplementation, and PERT treatment. Our results support that there was an imbalance in Ca, P, and Vit-D metabolism in these patients. In addition, there were significant associations between calcium, phosphorus, and vitamin D with several health and nutritional biomarkers. 

## Figures and Tables

**Table 1:** Baseline demographic and clinical characteristics of participants by type of mutation *ΔF508* (*n* = 17) [2,21,22].

Characteristics (Mean ± SD)	Total	Homozygous *ΔF508*	Compound Heterozygous *ΔF508*	*p*-Value
Age (years)	14. 8 ± 8	18.7 ± 10	12.7 ± 6.2	0.147
Triceps skinfold (mm)	8.5 ± 4.6	11.6 ± 5.7	6.8 ± 2.9	0.034 *
Polyunsaturated fats (%DRI)	17.1 ± 5.2	20.4 ± 5.6	15.1 ± 3.9	0.043 *
Vitamin C (%DRI)	170 ± 140	270 ± 159	110 ± 90	0.021 *
Vitamin D intake (%DRI)	623 ± 966	1259 ± 1281	243 ± 241	0.036 *
Iodine (%DRI)	53 ± 21	67 ± 16	45 ± 20	0.043 *
Transferrin (mg/dL)	258 ± 39	231 ± 26	274 ± 38	0.027 *
Blood urea nitrogen (mg/dL)	32.7 ± 11.3	41.5 ± 12.2	28.0 ± 7.8	0.013 *
Hemoglobin (g/dL)	14.3 ± 1.3	13.5 ± 1.0	14.9 ± 1.3	0.037 *
Leucocytes (cell/mm^3^)	7870 ± 1360	6877 ± 616	8466 ± 1349	0.017 *
Platelets (cell/mm^3^)	299 ± 103	224 ± 45	344 ± 103	0.018 *
IgG3 (mg/dL)	34.6 ± 22.2	18.4 ± 9.1	42.6 ± 22.6	0.043 *

Legend: DRI: %Dietary Reference Intake. * = *p* < 0.05

**Table 2:** Types of mutation in the compound heterozygous subgroup for *ΔF508* (*n* = 11).

Age (Years)	Type of Mutations
2	*1898 + 1G->A*
6	*1341 + 1G->A*
8	*711 + 1G->T*
8	*1717-1G->A*
9	*2183AA->G*
13	*G542X and Q890X*
15	*1777-1G*
15	*L997F*
16	*2183 AA->G*
20	*1341 + 1G->A and G673X*
23	*S549N*

**Table 3:** Genetics, clinical evaluation, dietary intake, and blood and urine analysis of calcium, phosphorus, and vitamin D in patients with cystic fibrosis (*n* = 17).

Age (Years)	Sex	BMI	Dietary Intake (%DRI)		Blood Analytical					Urine Analysis							
			Vit-D	Ca	Vit-D	Ca	P	Ca/P ratio	ALP	Calcium		Ca/Cr Ratio	Phosphorus		TRP %	TmP/ GFR	Ca/P Ratio
										mg/kg/d	24 hU		mg/kg/d	24 hU			
Homozygous																	
4	M	−0.59	**50**	109	**15.0**	9.9	5.80	1.69	**775**	1.20	22	0.02	33.93	621	**88.7**	5.4	0.61
9	F	−0.09	**69**	**137**	28.0	10.4	4.70	2.12	**555**	3.55	94	0.02	11.96	317	**87.9**	4.2	0.88
18	F	**−2.30**	2293	95	**15.0**	8.9	**3.80**	2.26	**128**	2.86	105	0.07	29.32	1076	**91.0**	3.8	1.17
23	F	−0.02	159	114	**11.0**	9.4	4.10	**2.27**	**129**	3.89	235	**0.53**	13.70	828	78.8	3.2	1.38
25	F	0.64	2574	**147**	26.0	9.4	4.10	**2.27**	**130**	1.59	92	0.08	13.41	775	**90.8**	**4.1**	1.86
31	F	−1.85	2406	96	75.0	9.7	3.60	2.69	73	**4.50**	223	**0.62**	12.85	636	79.1	2.8	1.46
Heterozygous																	
2	M	−0.79	**35**	**147**	23.0	10.0	5.30	1.88	**550**	3.83	41	0.02	22.71	24	**90.9**	5.3	0.63
6	F	−1.16	**3**	**123**	**14.0**	9.9	4.60	2.13	442	1.24	22	0.03	17.68	313	85.5	3.9	0.75
8	M	−0.15	85	**267**	23.0	10.2	5.50	1.80	**595**	3.92	127	0.07	26.57	861	**91.1**	**5.5**	0.82
8	M	−0.62	**39**	**154**	22.0	9.6	**4.30**	2.23	**948**	1.64	51	0.03	22.79	709	**89.4**	3.9	1.32
9	F	−0.12	672	85	**17.0**	10.1	4.70	2.12	428	**6.35**	169	0.05	32.39	861	**89.5**	4.5	0.81
13	M	−0.52	**19**	109	24.0	9.7	5.00	1.94	**1071**	**4.74**	207	0.10	19.61	857	**93.4**	**5.4**	1.51
15	M	−1.66	99	**155**	**11.0**	9.9	5.30	1.87	**516**	3.40	69	0.05	30.86	1293	**89.7**	5.1	0.91
15	F	−1.72	**12**	103	20.7	10.6	**3.70**	2.97	**420**	1.79	72	0.06	17.54	705	80.7	2.9	0.98
16	F	**−3.81**	1425	87	**17.0**	10.2	4.50	2.22	**275**	**4.65**	165	0.06	16.24	575	90.5	**4.5**	1.66
20	F	−0.04	623	**127**	**7.0**	10.0	3.74	2.70	**187**	2.45	145	0.05	12.28	726	**92.1**	2.9	3.37
23	M	−1.35	**38**	96	**3.4**	9.5	3.90	2.44	113	3.22	115	0.12	20.74	712	79.7	3.1	1.26

Legend: BMI: body mass index. DRI: %Dietary Reference Intake. Vit-D: vitamin D. Ca: Calcium. P: phosphorus. ALP: alkaline phosphatase. Cr: Creatinine. TRP: fractional tubular reabsorption of phosphate. TmP/GFR: tubular maximum phosphate reabsorption per glomerular filtration rate. Abnormal values are written in bold.

**Table 4:** Regression analysis between nutritional parameters studied by calcium, phosphorus, and vitamin D levels in the diet and in blood and urine in patients with cystic fibrosis (*n* = 17).

Serum Calcium (mg/dL)	Serum Phosphorus (mg/dL)	Ca/Cr Ratio	Urine Ca (mg/dL)	Urine Phosphate (mg/dL)	Urine Ca/P Ratio	Calcium Excretion Rate	TRP (%)	TmP/GFR (mg/dL)
Linear	regression	analysis						
FM (kg) BIA *R*^2^ = 0.746 *p* = 0.006	Age (years) *R*^2^ = 0.562 *p* = 0.001			Indirect calorimetry *R^2^ =* 0.604 *p* = 0.001				Serum Ca/P ratio *R*^2^ = 0.839 *p* = 0.000
	Serum Ca/P ratio *R*^2^ = 0.838 *p* = 0.000		Basophiles *R*^2^ = 0.537 *p* = 0.001		MCHC *R^2^ =* 0.712 *p* = 0.000			Serum phosphorus *R*^2^ = 0.875 *p* = 0.000
Multilinear	regression	analysis						
		Creatinine *R*^2^ = 0.732 *p* = 0.000	FFM kg A *R*^2^ = 0.537 *p* = 0.009		Hip C *R*^2^ = 0.510 *p* = 0.002	Creatinine *R*^2^ = 0.722 *p* = 0.000		Creatinine *R*^2^ = 0.588 *p* = 0.000
		CER *R*^2^ = 0.999 *p* = 0.000	Vitamin B12 (%DRI) *R*^2^ = 0.555 *p* = 0.001		FFM kg A *R*^2^ = 0.569 *p* = 0.001			
		CD3 T L *R*^2^ = 0.631 *p* = 0.000	CD19 T L *R*^2^ = 0.638 *p* = 0.000			CD3 T L *R*^2^ = 0.621 *p* = 0.000	IgM *R*^2^ = 0.616 *p* = 0.000	
			BUN and GGT *R*^2^ = 0.586 *p* = 0.002					

Legend: Ca: Calcium. P: phosphate. Cr: Creatinine. TRP: fractional tubular reabsorption of phosphate. TmP/GFR: tubular maximum phosphate reabsorption per glomerular filtration rate. %DRI: %Dietary Reference Intake. MCHC: mean corpuscular hemoglobin concentration. FFM: fat-free mass. kg: kilograms. BIA: bioelectrical impedance analysis. A: anthropometry. C: Circumference. CER: Calcium excretion ratio. L: lymphocytes. BUN: blood urine nitrogen. GGT: gamma-glutamyl transferase.

## Data Availability

Data are contained within the article and Supplementary Materials. Data supporting the reported results can also be found in references [2,21,22].

## Supplementary Materials

The following supporting information can be downloaded at https://www.mdpi.com/article/10.3390/ijms25031900/s1, Table S1: Significant correlations in the entire series between the nutritional parameters studied with the levels of calcium, phosphorus and vitamin D in the diet, blood and urine.

## Abbreviations

CF	Cystic fibrosis
Ca	Calcium
P	Phosphorus or phosphate
Vit	Vitamin
PA	Physical Activity
RI	Respiratory insufficiency
RS	Respiratory sufficiency
PI	Pancreatic insufficiency
PS	Pancreatic sufficiency
BMI	Body mass index
%DRI	% Dietary Reference Intake
ALP	Alkaline phosphatase
Cr	Creatinine
TRP	Fractional tubular reabsorption of phosphate
TmP/GFR	Tubular maximum phosphate reabsorption per glomerular filtration rate
EE	Energy expenditure
ECFS	European Cystic Fibrosis Society
ESPEN	European Society for Clinical Nutrition and Metabolism
ESPGHAN	European Society for Paediatric Gastroenterology Hepatology and Nutrition
WHO	World Health Organization
NB	Nitrogen balance
CRP	C-reactive protein
ESR	Erythrocyte sedimentation rate
NHANES	National Health and Nutrition Examination Survey
PERT	Pancreatic enzyme replacement therapy
FAC	Fat absorption coefficient
FFM	Fat free mass
FM	Fat mass
BIA	Bioelectrical impedance analysis
