# Supplementary material for: Calcium, Phosphorus, and Vitamin D Levels in a Series of Cystic Fibrosis Patients: A Cross-Sectional Study
Source: Int J Mol Sci. 2024 Feb 5;25(3):1900. doi: 10.3390/ijms25031900 (PMC10856093; doi:10.3390/ijms25031900)
Supplement: Supplementary file 1 [file ijms-25-01900-s001.zip › ijms-2769014-supplementary.pdf]

International Journal of *Molecular Sciences*

MDPI

*Supplementary Material*

# Calcium, Phosphorus, and Vitamin D Levels in a Series of Cystic Fibrosis Patients: A Cross-sectional Study

**Marlene Fabiola Escobedo-Monge [1,*], Marianela Marcos-Temprano [2], Joaquín Parodi-Román [3], María Antonieta Escobedo-Monge [4], Carmen Alonso-Vicente [5], María Carmen Torres-Hinojal [1] and José Manuel Marugán-Miguelsanz [5]**

1 Faculty of Medicine, University of Valladolid, Avenida Ramón y Cajal, 7, 47005 Valladolid, Spain; mctorresh@telefonica.net
2 Castilla y León Cystic Fibrosis Unit, University Clinical Hospital of Valladolid, Avenida Ramón y Cajal, 3, 47005 Valladolid, Spain; marianela_mt6@hotmail.com
3 Science Faculty, University of Cadiz, Paseo de Carlos III, 28, 11003 Cádiz, Spain; joaquin_parodi@yahoo.es
4 Department of Chemistry, Science Faculty, University of Burgos, Plaza Misael Bañuelos sn, 09001 Burgos, Spain; antoitalia777@gmail.com
5 Department of Pediatrics of the Faculty of Medicine, Valladolid University; Section of Gastroenterology and Pediatric Nutrition, University Clinical Hospital of Valladolid, Avenida Ramón y Cajal, 7, 47005 Valladolid, Spain; carmenalonso@gmail.com (C.A.-V.); jmmarugan@telefonica.net (J.M.M.-M.)
* Correspondence: amescobedo@msn.com; Tel.: +34-639-590-467

**Table S1.** Significant correlations in the entire series between the nutritional parameters studied with the levels of calcium, phosphorus and vitamin D in the diet, blood and urine (*n* = 17).

| | Vitamin D intake | | Serum phosphorus | | Serum Ca/P ratio | | Urine Ca/P ratio | |
|---|---|---|---|---|---|---|---|---|
| | *r* | *p-value* | *r* | *p-value* | *r* | *p-value* | *r* | *p-value* |
| Age (years) | | | -0.776** | 0.000 | 0.761** | 0.000 | | |
| Weight-for-age | | | | | 0.637** | 0.006 | 0.829** | 0.000 |
| Height-for-age | | | -0.780** | 0.000 | 0.798** | 0.000 | 0.788** | 0.000 |
| Wrist circumference | | | | | | | 0.705** | 0.002 |
| Waist circumference | | | | | | | 0.806** | 0.000 |
| Hip circumference | | | -0.659** | 0.006 | 0.677** | 0.004 | 0.874** | 0.000 |
| Subscapular skinfold | 0.658** | 0.006 | | | | | 0.658** | 0.006 |
| FM by anthropometry | | | | | | | 0.626** | 0.009 |
| FM kg by anthropometry | | | | | | | 0.806** | 0.000 |
| FFM by anthropometry | | | | | | | -0.626** | 0.009 |
| FFM kg by anthropometry | | | -0.675** | 0.003 | 0.702** | 0.002 | 0.853** | 0.000 |
| FM by BIA | | | | | 0.721** | 0.002 | | |
| FFM by BIA | 0.664** | 0.007 | | | | | 0.729** | 0.001 |
| FFM kg by BIA | | | | | | | 0.857** | 0.007 |
| MUAC | | | | | | | 0.693** | 0.003 |
| MAMC | | | | | | | 0.746** | 0.001 |
| Arm area | | | | | | | 0.693** | 0.003 |
| Arm muscle area | | | | | | | 0.717** | 0.002 |
| Cholesterol (mg/day) | | | -0.668** | 0.005 | | | | |
| BUN (mg/dL) | 0.631** | 0.009 | | | | | | |
| TmP/GFR | | | | | -0.916** | 0.000 | | |
| C-reactive protein | -0.567** | 0.044 | | | | | | |
| Serum Ca/P ratio | | | -0.977** | 0.000 | | | | |
| Creatinine | | | -0.734** | 0.001 | | | | |
| Glomerular filtration rate | | | 0.682** | 0.003 | -0.675 | 0.003 | | |
| Alanine aminotransferase | | | 0.606** | 0.010 | | | | |
| Alkaline phosphatase | | | 0.776** | 0.000 | | | | |
| Duration disease | | | -0.631** | 0.007 | **Serum Vitamin D** | | | |
| MCH | 0.689** | 0.005 | | | *r* | *p-value* | | |
| MCHC | | | | | 0.778** | 0.001 | -0.844** | 0.000 |
| Urine phosphorus | | | 0.600 | 0.014 | | | | |
| Urine Ca/P ratio | | | -0.562* | 0.023 | | | | |
| Basophil | -0.651** | 0.009 | | | | | | |

| | Serum calcium | | Calcium intake | | Urine calcium | | Ca/Cr ratio | |
|---|---|---|---|---|---|---|---|---|
| | *r* | *p-value* | *r* | *p-value* | *r* | *p-value* | *r* | *p-value* |
| FM kg by BIA | -0.886** | 0.003 | | | | | | |
| FFM by anthropometry | | | 0.683** | 0.004 | | | | |
| Suprailiac Z-score | | | 0.646** | 0.007 | | | | |
| Urine nitrogen | -0.654** | 0.008 | | | | | | |
| Serum zinc | | | 0.679** | 0.004 | | | | |
| Calcium/magnesium ratio | | | 0.641** | 0.007 | | | | |
| Vitamin 12 (%DRI) | | | | | 0.751** | 0.001 | | |
| Alkaline phosphatase | | | | | | | -0.607 | 0.010 |
| BUN (mg/dL) | | | | | 0.748** | 0.001 | | |
| GGT | | | | | 0.748** | 0.001 | | |
| Basophils | | | | | -0.762** | 0.001 | | |
| CD3 T Lymphocytes | | | | | | | 0.788** | 0.000 |
| CD19 T Lymphocytes | | | | | -0.839** | 0.000 | | |
| Urine Ca/P ratio | | | | | 0.745** | 0.001 | | |
| Calcium/creatinine ratio | | | | | 0.745** | 0.001 | | |
| Creatinine | | | | | | | 0.763** | 0.000 |

Legend: Ca: calcium, P: phosphorus o phosphate. Cr: creatinine. FM: fat mass, FFM: fat-free mass. Kg: kilograms. BIA: bioelectrical impedance analysis. MUAC: mid upper-arm circumference. MAMC: mid arm muscle circumference. BUN: blood urine nitrogen. TmP/GFR: Tubular maximum phosphate reabsorption per glomerular filtration rate. %DRI: %Dietary Reference Intake. GGT: Gamma-glutamyl transferase. MCH: mean corpuscular hemoglobin. MCHC: mean corpuscular hemoglobin concentration. * $p < 0.05$. ** $p < 0.01$.
